# Clear cell carcinoma of the ovary: a retrospective multicentre experience of 254 patients with complete surgical staging

**DOI:** 10.1038/sj.bjc.6603116

**Published:** 2006-04-25

**Authors:** M Takano, Y Kikuchi, N Yaegashi, K Kuzuya, M Ueki, H Tsuda, M Suzuki, J Kigawa, S Takeuchi, H Tsuda, T Moriya, T Sugiyama

**Affiliations:** 1 Department of Obstetrics and Gynaecology, National Defence Medical College, Tokorozawa, Saitama 359-8513, Japan; 2 Department of Obstetrics and Gynaecology, Tohoku University, Sendai, Miyagi 980-8574, Japan; 3 Department of Gynaecology, Aichi Cancer Center Hospital, Nagoya, Aichi 464-8681, Japan; 4 Department of Obstetrics and Gynaecology, Osaka Medical College, Takatsuki, Osaka 569-8686, Japan; 5 Department of Obstetrics and Gynaecology, Osaka City General Hospital, Toshima-ku, Osaka, Osaka 534-0021, Japan; 6 Department of Obstetrics and Gynaecology, Jichi Medical College, Kawachi-gun, Tochigi 329-0498, Japan; 7 Department of Obstetrics and Gynaecology, Tottori University, Yonago, Tottori 683-8504, Japan; 8 Department of Gynaecology, Kobe National Hospital, Kobe, Hyogo 554-0155, Japan; 9 Department of Pathology II, National Defence Medical College, Tokorozawa, Saitama 359-8513, Japan; 10 Department of Pathology, Tohoku University Hospital, Aoba-ku, Sendai 980-8574, Japan; 11 Department of Obstetrics and Gynaecology, Iwate Medical College, Morioka, Iwate 020-8505, Japan

**Keywords:** clear cell carcinoma, ovary, chemotherapy, paclitaxel, lymph node metastasis

## Abstract

A retrospective analysis was performed to evaluate the clinical characteristics and prognostic factors in the patients with clear cell carcinoma (CCC) of the ovary. After central pathological review and scanning of the medical records of nine Japanese institutions between 1992 and 2003, a total of 254 patients with CCC of the ovary were enrolled in the present study. Mean age was 52.4 years (range 23–73 years). Tumours were 13% (33/254) stage Ia, 36% (92/254) stage Ic, 13% (33/254) stage II, 30% (80/254) stage III, and 6% (16/254) stage IV. Five-year progression-free survival and overall survival was 84 and 88% in stage I, 57 and 70% in stage II, 25 and 33% in stage III and 0 and 0% in stage IV, respectively. Retroperitoneal lymph node metastasis was observed in 9% in pT1a tumours, 7% in pT1c tumours, 13% in pT2 tumours, and 58% in pT3 tumours, respectively. There was no survival benefit according to chemotherapeutic differences in the patients who received complete surgical staging procedures and conventional chemotherapy. Peritoneal cytological status was an independent prognostic factor in stage Ic patients (*P*=0.03) and only residual tumour diameter was an independent prognostic factor in stage III, IV patients (*P*=0.02). Our results suggest that cytoreductive surgery resulting in no residual tumour only could improve the prognosis of advanced CCC patients.

Cancer of the ovary has the worst prognosis of all gynaecological malignancies in the United States (Edwards *et al*, 2005) and Europe (Bray *et al*, 2005). Survival rate of patients with ovarian cancer has dramatically improved after introduction of platinum-based chemotherapy, but there still exist a large number of patients showing no response to the treatments. Although response to anticancer drugs is not easy to predict, *in vitro* studies suggested that acquired resistance to cisplatin has been associated with increased levels of glutathione and glutathione-S-transferase activity, increased metallothionein and decreased accumulation of cisplatin (Kikuchi *et al*, 1998). Histological subtypes such as clear cell carcinoma (CCC) and mucinous adenocarcinoma had been suggested as one of the most reliable criteria predicting the ineffectiveness of chemotherapy.

Clear cell carcinoma (CCC) was initially termed as mesonerhroid in 1939 (Schiller, 1939), and since 1973 it was strictly defined by World Health Organization as lesions characterised by clear cells growing in solid/tubular or glandular patterns as well as hobnail cells (Serov *et al*, 1973). Since then, many literatures have identified the distinctive behaviour of the tumors as compared with other histological subtypes of ovarian neoplasms. The most distinctive difference is that patients with CCC of the ovary have lower response rate to anticancer drugs. To our knowledge, only a few clinical studies have evaluated the response rates for CCC patients with measurable disease. The response rate of chemotherapy for CCC was 11.1% with platinum-based regimens (Sugiyama *et al*, 2000) and 22–56% with paclitaxel plus carboplatin. (Enomoto *et al*, 2003; Ho *et al*, 2004).

Another factor that might contribute to prognosis of ovarian cancer is the degree of cytoreductive surgery including lymphadenectomy. Complete surgical staging including para-aortic lymphadenectomy might influence the prognosis in early-stage CCC cases (Ho *et al*, 2003). Furthermore, the patients with pure-type CCC had worse overall survival than those with mixed-type CCC (Ho *et al*, 2004).

To evaluate the clinical characteristics of the patients with CCC of the ovary and to determine the impact of surgery and chemotherapy on prognosis of those patients, we conducted a retrospective study over 11-year period of a sample of 254 patients diagnosed with pure-type CCC in the departments of nine Japanese institutions.

## MATERIALS AND METHODS

### Patients and tumours

Between 1992 and 2002, 254 patients with CCC of the ovary were identified by scanning the medical records of the collaborating institutions and central pathological review. Patients received initial treatment and follow-up at nine institutions belonging to Japan Clear Cell Carcinoma Study Group; National Defence Medical College Hospital, Tohoku University Hospital, Aichi Cancer Center Hospital, Osaka Medical College Hospital, Osaka City General Hospital, Jichi Medical College Hospital, Tottori University Hospital, Kobe National Hospital, Iwate Medical College Hospital.

Initially, 337 patients were accrued from medical records of each institution. All pathological specimens from primary surgery were reviewed under central pathological review by two independent pathologists with no knowledge of patients' clinical data. Tumours were diagnosed as CCC if typical clear or hobnail cells growing in a papillary, solid, or tubulocystic pattern appeared in >90% of all pathological specimens. After pathological review, three cases were excluded; two diagnosed as mixed epithelial ovarian cancers and the other diagnosed as CCC derived from mature cystic teratoma, and 334 cases were identified as the patients with pure-type CCC of ovary. In those patients, 80 patients were excluded owing to insufficient surgery lacking complete surgical staging procedures: 13 cases in pT1a tumours, 51 cases in pT1c tumours, 16 cases in pT2 tumours, respectively. The rest 254 patients were enrolled on the present study. Patients of FIGO stage Ic were classified into three subtypes according to pathological characteristics; Ic (capsule ruptured) for the patients with ruptured capsule at laparotomy, Ic (ovarian surface) for those with tumour on ovarian surface, and Ic (ascites/malignant washing) for those with positive malignant cells in the ascites or positive peritoneal washing.

All 254 patients underwent complete surgical staging procedures including hysterectomy, bilateral salpingo-oophorectomy, peritoneal washing, omentectomy, pelvic lymphadenectomy and para-aortic lymphadenenctomy. Staging was based on the FIGO classification. The resected lymph node counts were not considered for the completion of the lymphadenectomy. A pN1 case was determined as having one or more lymph node metastasis in pelvic or paraaortic lymph nodes.

### Chemotherapy

Two hundred and forty-two (95.3%) patients received postoperative chemotherapy after initial surgery. Second look operation or second reductive surgery was done by surgeon's preference. Combination therapy of cyclophosphamide and doxorubicin and cisplatin (CAP) was as follows: one cycle consisted of a drip infusion of 50–75 mg m^−2^ cisplatin for 3 h accompanied by an i.v. injection of 50 mg m^−2^ doxorubicin and 500 mg m^−2^ cyclophosphamide and six cycles were given every 4 weeks. Paclitaxel and platinum regimen consisted of an infusion of 175–180 mg m^−2^ of paclitaxel and 50–75 mg m^−2^ of cisplatin or carboplatin (AUC=5–6). Other regimens included the combination chemotherapy irinotecan hydrochloride and cisplatin (40 cases) and irinotecan hydrochloride and mitomycin C (20 cases) and irinotecan hydrochloride and etoposide (3 cases). One cycle of irinotecan hydrochloride and platinum regimen consisted of a drip infusion of 50–60 mg m^−2^ of cisplatin on day 1 and 50–60 mg m^−2^ of CPT-11 on day 1, 8, 15 and 1 week off and it was repeated every 4 weeks.

Response was evaluated with CT or MR images for patients with measurable disease. A complete response (CR) was defined as the complete disappearance of all detectable disease for at least 4 weeks. A partial response (PR) was defined as a >50% decrease in tumour size for at least 4 weeks. Stable disease (SD) was defined as the absence of any significant change in measurable lesions for at least 4 weeks. Progressive disease (PD) was defined as the appearance of a new lesion or a >25% increase in tumour size. Serum levels of tumour markers including CA125 were not used for response evaluation of chemotherapy in the present study.

The time to progression was defined as the interval from the date of primary surgery until the date of recurrence or tumour progression (PD). Survival duration was determined as the time from the date of primary surgery until death or the date of last follow-up contact.

### Statistical methods

Kaplan–Meier method was used for calculation of patient survival distribution. The significance of the survival distribution in each group was tested by a generalized Wilcoxon test and the log-rank test. The *χ*^2^-test and Student's *t*-test for unpaired data were used for statistical analysis. A *P*-value of <0.05 was considered statistically significant. The Stat View software ver.5.0 (SAS Institution Inc., Cary, NC, USA) was used to analyse the data.

## RESULTS

### Patients and tumours

The characteristics of the study population are summarized in Table 1. Mean age was 52.4 years (range 23–73 years). Tumours were 13% (33/254) stage Ia, 36% (92/254) stage Ic, 13% (33/254) stage II, 31% (80/254) stage III, and 6% (16/254) stage IV, respectively. There is no case with stage Ib tumours. Among 92 cases of stage Ic, there were 45 cases (49%) of Ic (capsule ruptured), 3 cases (3%) of Ic (ovarian surface) and 44 cases (48%) of Ic (ascites/malignant washing), respectively. In 75 stage IIIc tumours, 15 cases (20%) were upstaged to stage IIIc because of retroperitoneal lymph node metastasis and 20 patients (27%) had both retroperitoneal lymph node metastasis and intra-peritoneal disease. Residual tumour diameter after primary debulking surgery was 0 cm in 176 cases (69%), less than 1 cm in 18 cases (7%), and more than 1 cm in 60 cases (24%), respectively.

Postoperative chemotherapy was offered for all patients, and 242 patients (95%) received anticancer drugs. Eight patients in stage Ia and four patients with stage Ic (capsule ruptured) refused postoperative chemotherapy.

Precise lymph node status according to pT distribution was documented in Table 2. Lymph node metastasis was documented in 3 of 36 patients (9%) in pT1a tumours, 7.1% in pT1c tumours, 13% in pT2, and 58% in pT3 tumours, respectively. Retroperitoneal lymph node metastasis in pT3 tumours was observed significantly more frequent than in pT1, 2 tumours (58.0 *vs* 8.7%, *P*<0.001, *χ*^2^-test).

### Response of chemotherapy

Response judged with CT or MRI images was assessable in 73 cases (29%) in 242 patients who received postoperative chemotherapy. Only 5 of 30 cases (16%) responded to CAP regimen. Progressive disease was documented in 23 patients (77%) and SD was observed in 2 patients (7%). In 28 patients treated with paclitaxel and platinum, response was observed in nine cases (32%) including one case with CR. In the patients treated with other regimens, response was observed in 3 of 10 patients (30%) treated with irinotecan hydrochloride and cisplatin. There is no responder in seven assessable patients who received combination with irinotecan hydrochloride and mitomycin C.

The median duration of progression-free survival for the patients with measurable disease was 4 months (range, 1–20 months) in CAP regimen, 5 months (range, 1–21 months) in paclitaxel and platinum, and 3 months (range, 2–20 months) in irinotecan hydrochloride and cisplatin, respectively.

### Clinical course

**Figure 1:**
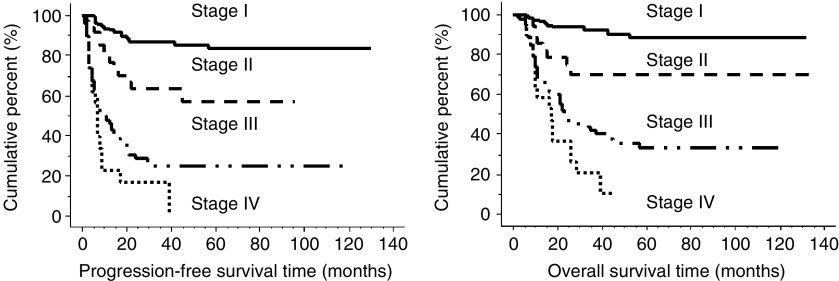
Progression-free survival and overall survival of patients depending on their FIGO stage. Five-year progression-free survival and overall survival was 84 and 88% in stage I, 57 and 70% in stage II, 25 and 33% in stage III and 0 and 0% in stage IV, respectively. *P*-values in progression-free survival were as follows: Stage I *vs* stage II, *P*<0.01; stage II *vs* stage III, *P*<0.01; stage III *vs* stage IV, *P*=0.35. *P*-values in overall survival were as follows: Stage I *vs* stage II, *P*<0.01; stage II *vs* stage III, *P*<0.01; stage III *vs* stage IV, *P*=0.17.

**Figure 2:**
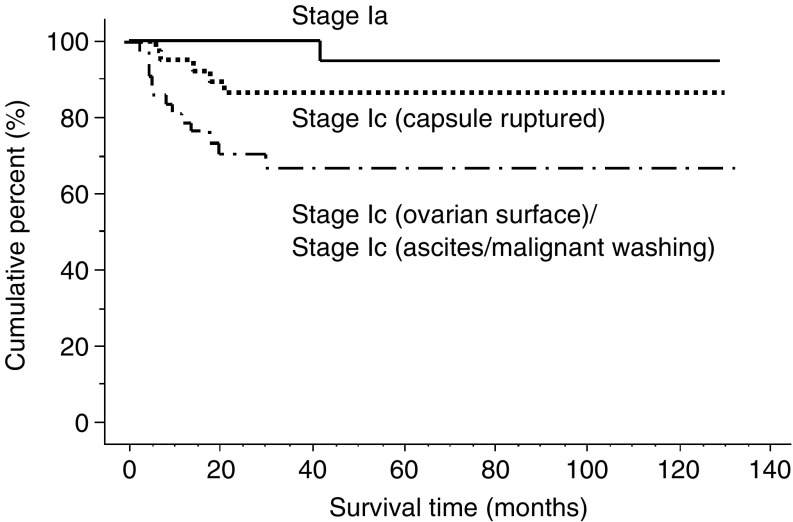
Progression-free survival of patients with FIGO stage I patients. There is no significant difference between patients with stage Ic (capsule ruptured) and those with stage Ia (*P*=0.11). Survival of the patients with stage Ic (ascites/malignant washing) and Ic (ovarian surface) was significantly worse than that of stage Ic (capsule ruptured) (*P*=0.04).

Average follow-up for all CCC patients in the present study is 47.4 months. Five-year progression-free survival and overall survival was 84 and 88% in stage I, 57 and 70% in stage II, 25 and 33% in stage III and 0 and 0% in stage IV, respectively (Figure 1). Although there is no statistically significant difference in progression-free survival between patients with stage Ic (capsule ruptured) and those with stage Ia (*P*=0.11), progression-free survival of the patients with stage Ic (ascites/malignant washing) and Ic (ovarian surface) was significantly worse than that of stage Ic (capsule ruptured) (*P*=0.04)(Figure 2). Multiple regression survival analysis for stage Ic patients with CCC revealed that positive peritoneal cytology was the only independent prognostic factor (*P*=0.03; Relative risk, 3.40; 95% CI, 1.14–10.18). Cumulative progression-free survival of pT1M0 patients with positive node was significantly lower than those with negative node (*P*<0.01). Five-year progression-free survival was 84% in pT1N0 patients and 56% in pT1N1 patients, respectively.

**Figure 3:**
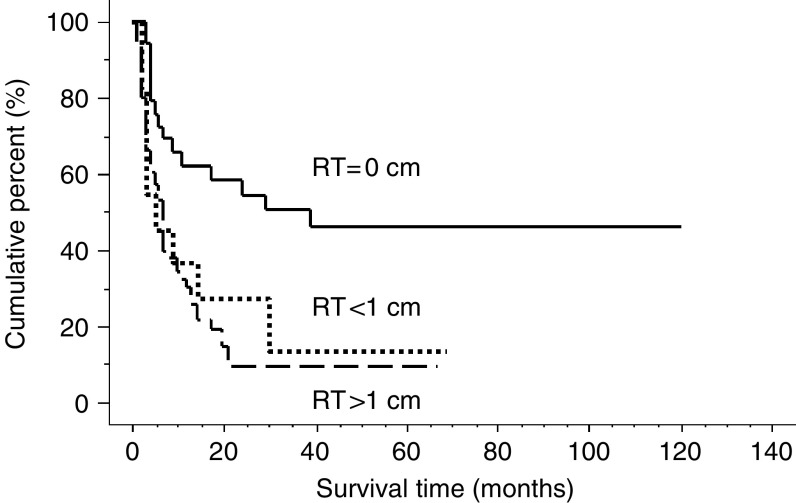
Progression-free survival of stage III, IV patients according to the residual tumour (RT) diameter. There is no significant prognostic difference between the patients with the tumour diameter less than 1 cm and those with the tumour diameter more than 1 cm (*P*=0.40). The patients with no residual tumour had significantly better progression-free survival than those with the tumour less than 1 cm (*P*=0.04) or those with tumour diameter more than 1 cm (*P*<0.01), respectively. Median progression-free survival duration was 39 months in the patients with no residual tumour, 7 months in those with the tumour diameter less than 1 cm, and 5 months in those with residual tumour diameter more than 1 cm, respectively.

Progression-free survival curves of stage III, IV patients according to the residual tumour diameter were shown in Figure 3. Median progression-free survival duration was 39 months in the patients with no residual tumour, 7 months in those with the tumor diameter less than 1 cm, and 5 months in those with residual tumour diameter more than 1 cm, respectively. There is no significant prognostic difference between the patients with the tumour diameter less than 1 cm and those with the tumour diameter more than 1 cm (*P*=0.40). The patients with no residual tumour had significantly better progression-free survival than those with the tumour less than 1 cm (*P*=0.04) or those with tumour diameter more than 1 cm (*P*<0.01), respectively.

Multiple regression analysis in stage III and IV patients revealed that chemotherapeutic regimen was not an independent prognostic factor (*P*=0.24) and only residual tumour diameter was an independent prognostic factor in stage III and IV patients (*P*=0.02) (Table 3).

## DISCUSSION

The present study and previous studies support that CCC of the ovary tended to present at earlier stages. Proportion of stage I/II tumours ranged from 59 to 71% (Yoonessi *et al*, 1984; Crozier *et al*, 1989; Jenison *et al*, 1989; Kennedy *et al*, 1989; O'Brien *et al*, 1993; Behbakht *et al*, 1998; Sugiyama *et al*, 2000). One of the reasons for the early detection was explained by the slow growing tumour behaviour (Itamochi *et al*, 2002a) and frequent presentation of the tumours as relatively large pelvic masses (Kennedy *et al*, 1989; Behbakht *et al*, 1998). In the present study, the status of peritoneal cytology was identified as an independent prognostic factor in FIGO stage Ic patients. Although tumour progression was observed in 5 (11%) of 45 stage Ic (capsule ruptured) tumours and one (3%) of 33 stage Ia tumours, there is no significant survival difference between two groups. Recent report analysing prognosis of early-staged ovarian cancer including only 25 CCC cases (26.6%) in 94 carcinomas showed no statistical significant difference between stages Ic preoperative *vs* intraoperative rupture (Leitao *et al*, 2004). Another report including higher ratio of CCC patients identified that stage Ic (capsule ruptured) patients showed significantly poorer survival than stage Ia patients (Mizuno *et al*, 2003). The present study implied the importance to remove the tumour mass without intraoperative rupture, especially in CCC patients.

Even in stage I ovarian cancer including all histological subtypes, the incidence of positive lymph nodes was not low, ranging from 5.1 to 20% (Sakuragi *et al*, 2000; Cass *et al*, 2001; Morice *et al*, 2003). It was reported that serous tumour had a higher incidence of lymph node involvement than non-serous tumors (Takeshima *et al*, 2005). Although the true incidence of lymph node metastasis in CCC tumour had not been clear, the present study revealed the frequency of metastasis in a large number of the CCC patients. Lymph node metastasis was observed in 3 of 36 patients (9.1%) in pT1a tumours, 7.1% in pT1c tumours, 10.8% in pT2 tumours, respectively. Fifteen (8.7%) of 173 patients who had pT1 or pT2 tumors were upstaged as stage IIIc tumours based on lymph node status. In general, prognostic significance of retroperitoneal lymph node metastasis in early-staged ovarian cancer patients was controversial. Survival rates with node-positive disease were significantly lower in clinical stage I and II disease (Kanazawa *et al*, 1999; Sakuragi *et al*, 2000; Negishi *et al*, 2004). In contrast, another report showed that the prognoses for clinical stage I/II patients with or without lymph node metastasis were similar (Onda *et al*, 1998). In pT1 CCC patients of the present study, lymph node status was identified as a strong prognostic factor and it is essential to accurately evaluate the lymph node status through complete surgical staging procedures. The study, called Adjuvant ChemoTherapy in Ovarian Neolasm (ACTION), revealed that no benefit of adjuvant chemotherapy was observed in early-stage ovarian cancer with optimal surgical procedures (Trimbos *et al*, 2003). In the present study, 12 patients with stage Ia or stage Ic (capsule ruptured) refused to receive chemotherapy, but there was no evidence of recurrence in median follow-up period of 44 months (range: 6–63 months), which might support the results of ACTION study.

Previous Japanese report have shown that the chemotherapeutic effect was assessable in only 27 patients (26.7%) in 101 CCC cases, in contrast it was assessable in 47% of serous adenocarcinoma (Sugiyama *et al*, 2000). In our series of CCC patients, patients with residual tumour diameter more than 1 cm were documented in only 60 (18%) of 254 cases, and the chemotherapeutic effect was assessable in only in 73 cases (29%) in 242 patients who received adjuvant chemotherapy. As the residual tumour after debulking surgery often lacked measurable tumour diameter to evaluate the effects of adjuvant chemotherapy in CCC patients, it has been quite difficult to select superior regimen.

There have been only a few reports to document the response of anticancer agents for CCC patients, but each of them included relatively small number of cases. The present study confirmed that CAP regimen showed a low response rate and quite a high incidence of PD in CCC patients as described previously (Sugiyama *et al*, 2000). The combination chemotherapy consisting of paclitaxel and platinum has been established as standard therapy for ovarian cancer. One report of paclitaxel and platinum regimen for CCC patients revealed that the response was observed in two of nine cases (22%) (Enomoto *et al*, 2003), and the other report of paclitaxel plus platinum chemotherapy showed the response was observed in 9 of 15 cases (56%) (Ho *et al*, 2004). These two studies including the present study suggested that paclitaxel plus platinum regimen had higher response rate compared to platinum-based chemotherapy. One report showed survival benefit of conventional chemotherapy with paclitaxel and platinum after complete surgery in CCC patients (Ho *et al*, 2003). However, the results from our series of CCC patients showed that there was no survival benefit with chemotherapy with paclitaxel and platinum compared with CAP regimen in both early and advanced cases. Irinotecan hydrochloride was preliminary introduced for CCC patients in clinical settings (Shimizu *et al*, 1998; Adachi *et al*, 1999; Kita *et al*, 2000), but there is no large clinical trial for the treatment of CCC patients of the ovary. Further studies are needed to establish the candidate regimen for CCC of the ovary.

Recent studies have suggested that CCC tumour showed a distinctive molecular behaviour from other histological subtypes. *In vitro* study suggested that paclitaxel and irinotecan hydrochloride were the candidates for anti-neoplastic agents for CCC (Itamochi *et al*, 2002b), but the present study has failed to prove the survival benefit of these two drugs in CCC patients. Another strategy for CCC tumours might be the additive use of molecular targeting agents. It was reported that hepatocyte nuclear factor-1 beta (HNF-1 β) was a CCC-specific marker and had anti-apoptotic effects in CCC cell lines (Tsuchiya *et al*, 2003). Another candidate marker could be ABCF2, which belongs to the ATP-binding cassette gene superfamily and is highly expressed in CCC and non-responders for chemotherapy (Tsuda *et al*, 2005). Suppression of CCC-specific molecular markers such as HNF-1 β or ABF2 may be another strategy for the treatment of CCC of the ovary. The present study clarified the significant prognostic importance of positive peritoneal cytology in early-stage CCC disease, and no macroscopic residual tumour in advanced CCC tumours, respectively. However, there was a little impact of chemotherapeutic effects on both early and advanced diseases. Although further studies are needed to identify effective agents in both anti-neoplastic agents and molecular targeting agents, our study provides the fundamental characteristics of CCC of the ovary.

## Figures and Tables

**Table 1:** Characteristics of the patients

**Characteristics**	**No. of patients (%)**
All cases	254
	
*Age (years)*	
<55	147 (57.9)
>55	107 (42.1)
	
*FIGO Stage*	
Ia	33 (13.0)
Ic (ovarian surface)	3 (1.2)
Ic (capsule ruptured)	45 (17.7)
Ic (ascites/malignant washing)	44 (17.3)
II	33 (13.0)
IIIa,b	5 (2.0)
IIIc	75 (29.5)
IV	16 (6.3)
	
*Residual tumour diameter*	
0 cm	176 (69.3)
<1 cm	18 (7.1)
>1 cm	60 (23.6)
	
*Postoperative chemotherapy*	
CAP^a^	76 (29.9)
Paclitaxel+platinum	103 (40.6)
Others	63 (24.8)
None	12 (4.7)

a CAP, cyclophosphamide+doxorubicin+cisplatin.

**Table 2:** Rates of lymph node metastasis according to pT status

**pT status**	**pN1**	**pN0**	**Rate of Lymph Node metastasis (%)**
pT1a (*n*=36)	3	33	9.1
pT1c (*n*=99)	7	92	7.1
pT2 (*n*=38)	5	33	13.1
pT3 (*n*=81)	47	34	58.0
			
Total (*n*=254)	62	192	24.4

**Table 3:** Multiple regression survival analysis for stage III, IV patients with CCC

**Variables**	**Hazard ratio**	**95% confidence interval**	***P***
*Age (years)*			0.96
<54	1		
>55	0.99	0.60; 1.61	
			
*PS*			0.67
0	1		
1,2	1.06	0.79; 1.43	
			
*FIGO stage*			0.22
III	1		
IV	1.47	0.80; 2.70	
			
*Residual tumour*			0.02
None	1		
<1 cm	2.23	0.89; 5.54	
>1 cm	3.17	1.68; 6.00	
			
*Chemotherapy*			0.24
CAP^a^	1		
Paclitaxel+platinum	0.56	0.48; 1.88	
Others	0.95	0.32; 1.22	

a CAP, cyclophosphamide+doxorubicin+cisplatin.
